# Essential Domain-Dependent Roles Within Soluble IgG for *in vivo* Superantigen Properties of Staphylococcal Protein A: Resolving the B-Cell Superantigen Paradox

**DOI:** 10.3389/fimmu.2018.02011

**Published:** 2018-09-19

**Authors:** Alejandro J. Ulloa-Morales, Carl S. Goodyear, Gregg J. Silverman

**Affiliations:** ^1^Laboratory of B-cell Immunobiology, Department of Medicine, New York University School of Medicine, New York, NY, United States; ^2^Institute of Infection, Immunity and Inflammation, College of Medical, Veterinary and Life Sciences, University of Glasgow, Glasgow, United Kingdom

**Keywords:** B cells, superantigens, protein A, antibodies, host-pathogen, anaphylaxis, SpA MRSA

## Abstract

*Staphylococcus aureus* is a common commensal and frequent opportunistic pathogen that causes invasive infections that often recur. Co-evolution with the host has led to the development of toxins that affect diverse immune cell types. Recent reports have highlighted the contributions of staphylococcal protein A (SpA). This small oligomeric secreted protein contains 4–5 homologous domains with two distinct immunoglobulin-binding sites; one for IgG Fc domains, while a separate site binds an evolutionarily conserved surface on Fab encoded by VHIII clan related genes. The Fab-binding site has been implicated in *in vivo* supraclonal VHIII-BCR targeted B-cell depletion by an activation induced death pathway. Yet the concept of a superantigen for B lymphocytes poses a seeming paradox. Unlike TCR that are expressed only in a membrane-associated form, BCR are expressed in both a membrane BCR form and in secreted Ig forms, which permeate virtually every part of the body at high levels. We therefore asked, why circulating immunoglobulin do not block the superantigen properties of SpA? Herein, we show that soluble IgG molecules are not *in vivo* inhibitors of these B-cell superantigen effects but are instead essential for potentiating these properties. We also show that the Fc subclass of circulating IgG is an indirect critical determinant of the B-cell superantigen effect. In contrast, host FcγR and complement are not required for SpA mediated *in vivo* B-cell depletion. Unexpectedly, after VHIII-IgG2a pretreatment SpA challenge resulted in fatal anaphylactic reactions, which we speculate may have involved FcγR interactions with mast cells and basophils. Cumulatively, our findings illuminate a cunning and potent molecular strategy by which a bacterial toxin effectively confounds the contributions of host B-lymphocytes to immune defenses.

## Introduction

*Staphylococcus aureus* is estimated to cause over half a million cases of invasive infection, with more than 10,000 deaths annually in the United States, in some years exceeding those attributed to influenza, viral hepatitis, and HIV/AIDS together (1, 2). *S. aureus* is also a ubiquitous commensal, with an estimated 30% of the population continuously colonized (3) and intermittent colonization of many more (4). Antibiotic-resistant strains, including methicillin-resistant *S. aureus* (MRSA), are increasing causes of community-acquired as well as hospital-acquired infections (5). Invasive infections also frequently recur (6) as prior bacteremia or skin infections generally do not reinforce host immune defenses (7–10).

*S. aureus* has a remarkable ability to evade host defenses through release of virulence factors, which can impair and/or deplete a range of different cell types (11–13). Even though functional antibodies and the complement system are known to play critical roles in defense (14, 15), *in vivo* experimental models have shown that mice with competent B cells and antibody responses can fare worse in containing and clearing *S. aureus* infection than do B-cell deficient mice (16–18).

Staphylococcal protein A (SpA) is a virulence factor consistently found in clinical isolates (19). With secretion during infection, cleavage of the signal peptide leads to linkage of the mature SpA polypeptide, via the amino-terminal X domain, to the cell wall bacterial peptidoglycan (20, 21) that tethers the 4-5 highly homologous Ig-binding domains of 56–61 amino acids that each fold into triple alpha helical bundles in tandem (22, 23). Co-evolution with the host immune system has imparted this toxin with functional capacities for Ig-binding that are highly conserved in different clinical isolates with only minor sequence variations in encoding *spa* genes (19).

During the pathogenesis of infection, SpA is postulated to inhibit the capacity of host antibodies and leukocytes for the opsonophagocytic killing (OPK) of *S. aureus* (24). Indeed, when immersed in human or mouse blood, staphylococci are immediately coated with Ig (25), which contributes to escape of this microbe from OPK by neutrophils and macrophages (26, 27). *In vivo* SpA exposure can significantly impair immune responses to bacterial antigens (28, 29), and has also been implicated in the impairment of anamnestic responses that would otherwise prevent reinfection (18, 30), although the responsible mechanism is not understood.

The Ig-binding domains of SpA have a site for interactions with IgG-Fc domains that has been credited with the above-described capacity of SpA to impair OPK function (31–33) (Supplemental Table 1). These homologous SpA domains also have a Fab-binding site that facilitates non-immune binding interactions with a large proportion (~30%) of human peripheral B cells (34–36) as well as 5–10% of mature murine B cells in immunologically naïve individuals (37). These interactions result from recognition of a conserved conformational surface on VH regions encoded by structurally related genes of the VHIII clan that are highly represented in almost all mammalian immune systems (34, 35, 37–40). The non-immune Fab interactions with SpA are therefore fundamentally distinct from immune recognition by lymphocytes of a specific conventional antigen, which generally interact with no more than 0.001 to 0.1% of the naïve repertoire [discussed in (41)].

Indeed, the SpA toxin-BCR co-complex displays many structural and functional similarities with those of known microbial T-cell superantigens (40, 41). Importantly, *in vivo* exposure to SpA is known to initially induce proliferation of large supra-clonal VHIII-restricted sets of B cells, by a process that can be transiently enhanced by second signal (i.e., co-stimulation) influences from cytokines, T helper cells, or bacterial factors, presumably also by staphylococcal peptidoglycans (42). Nonetheless, *in vivo* exposure ends in large scale VH-targeted activation-induced cell death (AICD) (28, 43–46).

This pathway is initiated by cellular internalization of the BCR-SpA complex, with concurrent down-modulation of CD19 and CD21 co-receptors (43). These targeted B cells then up-regulate activation markers (43), depolarize their mitochondrial membranes (43), and display induction of markers of apoptotic death, including the BH3-only Bcl2 member, Bim (46) and activate effector caspases (43, 44). Apoptotic B cells are then deposited in peripheral lymphoid tissues (43). SpA induced VH selective *in vivo* B-cell depletion has also been documented at a repertoire level (43) in a process that progresses to completion by 72 h if second signal is limiting [reviewed in (41)]. Earlier reports also demonstrated the dose-dependent B-cell depletion effect of *in vivo* SpA challenge doses, from 2 μg to 2 mg (i.e., 0.0476–47.6 pmol) (45), and the finding that SpA-mediated B-cell depletion has an absolute requirement for circulating Ig (43). Yet the contributions of different subdomains within IgG has been unclear, especially as SpA has both Fc- and Fab-binding domains.

Structural analysis of a SpA-BCR co-complex has revealed many parallels with known microbial T cell superantigens [reviewed in (41)]. However, from this perspective the nature of the superantigen properties of SpA (41-46) also presents an inherent paradox. Unlike T cells that solely express their antigen receptors as membrane-associated structures, BCR exist in two forms: as lymphocyte membrane-associated receptors; and as secreted Ig that diffuse into virtually every part of the body. Whereas SpA can interact with VHIII-Fab BCR on B cells and with the same sites on soluble Ig, it has been unclear how the *in vivo* properties of SpA, mediated by the binding of VH targeted B cells, are not in competition with the stoichiometrically superior numbers of circulating Ig molecules that also permeate the tissues.

Yet, there are gaps in our understanding of the structural requirements for *in vivo* superantigen responses to SpA. Infusions of a labeled form of SpA were shown to result in BCR-mediated binding interactions with targeted B cells (43, 44), while in much earlier studies Langone and coworkers, showed that following intravenous infusion, SpA persists for more than 24 h in the circulation in dynamically changing heterogeneous complexes with circulating Ig, with the most common form composed of one oligomeric SpA molecule and two IgG molecules (47–50). Yet in these reports, the role of the Fab-binding site of SpA was not considered.

In the present study, we elucidated how B-cell depletion by this archetypical B-cell superantigen can target B cells *in vivo* within a milieu saturated with high concentrations of soluble IgG. In fact, our studies considered the potential contributions of the two distinct types of Ig-binding sites on oligomeric SpA molecules, as well as for involvement of host IgG triggered effector functions that are integral to host defenses. Our findings demonstrate that soluble IgG clearly is not an inhibitor of the superantigen properties of SpA, but instead serves as an essential potentiator.

These findings suggest a model of functional cooperativity between *in vivo* membrane BCR and circulating IgG that rationalizes the *in vivo* properties of known B-cell superantigens. Our results therefore explain how the interactions between SpA and host BCR/antibodies fully arm this opportunistic pathogen with potent countermeasures for subversion of host defenses.

## Methods and materials

### Immunoglobulins

Recombinant murine VHIII IgG1, IgG2a or IgG3 were generated with the variable regions of the anti-PC TEPC15 parental clone (51), while the VHI isotype control IgG express antibody genes from the anti-Hen Egg Lysozyme (HEL) D1.3 mAb (52), which were generated by transfection of CHO cells and subsequent cloning and expansion, using the OptiVEC system (Invitrogen, Thermo Fischer). Antibody gene sequences of constructs were confirmed by automated sequencing (not shown). Cell populations were expanded using the WAVE Bioreactor (GE Healthcare) with serum-free medium, with subsequent concentration and sterile filtration, with purity confirmed by PAGE and HPLC (AKTA FPLC, GE Healthcare). All preparations were effectively devoid of aggregates (HPLC <<1%) and were without detectable endotoxin, by Limulus assay (Lonza). Concentrations were determined by BCA (Sigma) and ELISA. Samples were stored at −80°C until used.

### Mouse strains

T15i (B6.129P2-Igh^tm1Cgn^) “knock-in” mice were used to provide B cell targets as these express a rearranged immunoglobulin heavy chain variable (VH) region from a hybridoma expressing a fixed S107.1 (VHIII clan) rearrangement of the TEPC15 clone inserted into the heavy chain locus in its natural position, replacing the J_H_ elements. These Heavy chains, which bear the IgM“^a^” allotype, are expressed with pairing with wild-type polyclonal light chains (kind gift of Prof. Klaus Rajewsky)(53). These mice have been backcrossed with C57BL/6 mice. C57BL/6 and congenic B-cell deficient muMT, Fcεr1g^tm1Rav^, C3(-/-) mice (54) were obtained from Taconic, and C1q -/- mice (55) were the kind gift of Peter Henson and Marina Botto, then bred and housed under pathogen-free conditions with water and food *ad libitum*. Animal studies were supervised by the NYU School of Medicine IACUC. Animals were immunophenotyped by flow cytometry, as previously described (43). In all experiments, mice were used at 8 to 14 weeks of age.

### *In vitro* stimulation studies

Adapting earlier methods (43, 56), T15i splenic murine mononuclear cells were isolated and purified, and viable cells were loaded with Fluo-4 (Invitrogen), as per manufacturer's protocol. Under replicate conditions, B cells were suspended in complete RPMI supplemented with 2-mercaptoethanol and L-glutamine and incubated without or with goat IgG Fab'2 anti-murine Ig (Jackson Immunoresearch) or recombinant SpA (Repligen) at 10 μg/ml in the absence or presence of serum murine IgG at 200 μg/mL. Data were acquired with a FACSCalibur™ (Becton Dickinson) and analyzed with FlowJo™ software (Treestar).

### IgG binding immunoassays

Wells were coated overnight with 5 μg/ml of recombinant SpA (Repligen), or a chemically modified form of SpA (mSpA) that lacks Fc-binding activity but retains Fab-binding activity (35), mSpA, anti-mouse Ig light chain kappa (BioLegend), hen egg lysozyme (Sigma) or PC-4 BSA (Biosearch) in 100 mM carbonate buffer. Plates were blocked with 3% BSA in PBS. Samples were serially diluted, and reactivity assessed in duplicate over a range of concentrations. Polyclonal mouse IgG (Jackson ImmunoResearch) was used as positive control. For detection, we used biotinylated goat anti-murine kappa light chain-specific reagent (BioLegend) at 1:5000, and streptavidin-HRP (Jackson ImmunoResearch) at 1:10,000. Reactions were developed using TMB substrate (BioLegend) and stopped with phosphoric acid 1M. OD450 was quantified using an ELISA plate reader (Biotek).

### Adoptive cell transfer murine model

To evaluate the influence of circulating IgG on the outcome of SpA mediated B-cell depletion we used a previously described system (43, 46, 56, 57). Briefly, groups of recipient mice, either C57BL/6 (B6) or congenic B-cell and Ig-deficient muMT (53), in which each recipient received 1 mg in 1 ml of the VHIII IgG1, IgG2a or IgG3 i.p., or their respective VHI isotype controls or human polyclonal IgG (6.7 pmol) or human polyclonal IgG Fab'2 (9.1 pmol)(Jackson ImmunoResearch). Then, after 24 h we infused CFSE-labeled dissociated mononuclear splenic cells from heterozygotic T15i mice. As previously described (43, 46, 56), splenic mononuclear cells were isolated from heterozygotic T15i mice, in which 20–30% of all B220-positive CD19-positive B cells express a BCR T15i VHIII clan transgene (IgM^a^ allotype bearing), while the remaining B-cells express a polyclonal wild-type BCR repertoire (IgM^b^ allotype bearing) (53). Splenocytes were pooled from different donors, then stained with CFSE (Invitrogen). Individual mice then received retro-orbital i.v. infusions of 25 × 10^6^ cells. Four hours later different groups received saline alone or 1 mg (23.8 pmol) of endotoxin-free rSpA by i.p. infusions, or as indicated. In each experiment, mice received an Ig treatment (unless specifically indicated), with groups that received saline or SpA infusions with 3 or more recipient mice per group. The efficiency of splenocyte adoptive transfer was not affected by specific congenic murine strain alone.

Based on the previously reported kinetics associated with completion of the *in vivo* BCR-mediated apoptotic death pathway (43), the spleens of recipient mice were harvested at 72 h after SpA challenge and individual flow analyses performed, as previously described (43, 46, 56). Serum samples documented IgG levels > 0.2 mg/mL in all harvested mice, by previously described assays (28). After infusion of VHIII Fab-IgG preparations, we confirmed the effective levels of anti-PC antibodies in the serum of recipients, by an in-house ELISA for IgG, and anti-PC binding activity, as previously described (28).

To investigate for a potential mechanism for the fatal reaction to co-treatments with SpA and VHIII-IgG2a, before challenge mice received intravenous treatment with Evans blue dye, which is used to detect increased vascular permeability associated with vascular injury (58).

### Flow cytometric analyses

To quantify the effect of SpA infusions on peripheral B cells, spleens were harvested 72 h post-SpA challenge and cell suspensions prepared, as described previously (43, 46, 56). Cells were maintained on ice throughout. For each mouse, 2 × 10^6^ cells were stained per panel in the presence of 20 μg/ml of Fc block (Pharmingen, Becton Dickinson) followed by staining with: B220-APC (eBioscience), CD3-PerCP Cy5.5 (BD), IgM^a^-PE (BD) or IgM^b^-PE (BD). For live/dead cell discrimination, we used fixable blue (Invitrogen) following the manufacturer's instructions. After staining, cells were washed and fixed in 4% paraformaldehyde. At least 5 × 10^5^ events per sample were collected on a LSR II flow cytometer (BD) running FACSDiva software. Data were analyzed with FlowJo (Treestar).

### Experimental infections and quantitation of SpA in abscesses

Adapting reported experimental infection methods (59), C57/BL6 or Ig-deficient μMT mice were infected with either *S. aureus* Newman or the isogenic *spa* knockout strain at 10^6^ CFU per animal by retro-orbital injection. At day 12 post-infection, mice were sacrificed and organs were harvested. Visible abscesses in liver and kidneys were dissected from the surrounding healthy tissue and weighed, mechanically macerated and digested with collagenase D (Sigma), and subsequently treated with DNase I. Each sample was boiled and centrifuged at 10,000 g for 10 min. Supernatants were evaluated at multiple dilutions for SpA using a commercial ELISA kit (Repligen). Abscesses produced by the Δ*spa* strain served as a negative control. Bacterial strains were the gift of Victor Torres, NYU.

### Complement depletion

Mice were i.p. treated 48 h before SpA challenge (1 mg/23.8 pmol, i.v.) with 30 μg cobra venom factor (CVF) (Quidel), which is twice the dose shown to completely deplete complement in C57/BL6 mice for up to a week after injection (60).

### Statistical analysis

Significant differences between groups were compared with Prism software (GraphPad) using unpaired student's *t* test (Mann-Whitney test), or as indicated. *p* < 0.05 was considered significant.

## Results

### Immunoglobulin complexed SpA induces B-cell activation

To investigate the potential influence of soluble IgG on SpA interactions with B cells via their membrane-associated BCR, we first performed a series of *in vitro* BCR targeted stimulation studies. For these investigations, we used B cells from T15i homozygotic transgenic knock-in mice, which express the VHIII clan-Fab associated SpA-binding motif on all B cells (28, 40) (see methods). Loading with Fluo-4 enabled detection of whether specific BCR engagement induces cellular calcium flux, as determined by release of this calcium ionophore.

**Figure 1:**
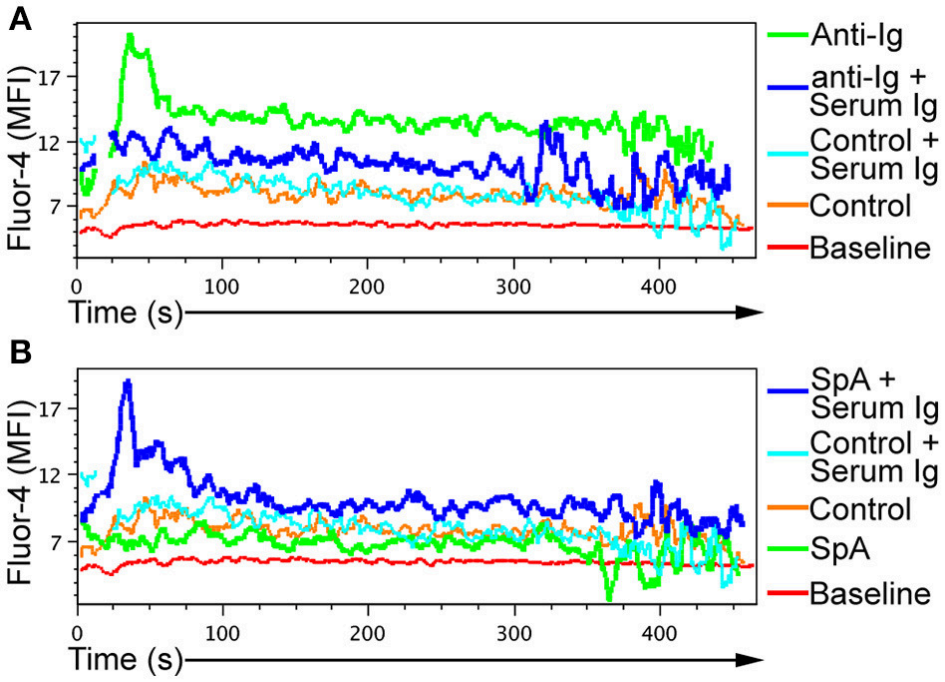
Induction of BCR-mediated *in vitro* B-cell activation by SpA requires soluble immunoglobulin. Splenic B cells from T15i mice were loaded with Fluor-4 dye, and calcium flux measured over time via flow cytometry. Cells were stimulated with either **(A)** goat Fab'2 anti-mouse Ig (H+L) (anti-Ig) or **(B)** soluble recombinant SpA, in the in the presence or absence of serum immunoglobulin (Ig), as indicated. For control conditions, cells were incubated in the absence of anti-Ig or SpA or soluble Ig. Assays results were repeated at least twice.

As a control for these studies, we used anti-Ig [as a F(ab)'2 preparation], which served as an experimental BCR ligand. This directly induced high-level calcium flux that reflected the BCR-mediated cell activation. As expected, incubation of the anti-Ig reagent along with soluble murine IgG substantially inhibited the BCR-mediated activation of these B lymphocytes that was demonstrated by greatly reduced calcium flux (Figure 1A).

In this culture system, SpA alone had little or no effect on B-cell calcium flux (Figure 1B). Yet in the presence of soluble IgG, SpA exposure instead induced marked calcium flux (Figure 1B). Taken together, these *in vitro* studies demonstrated that, unlike interactions with anti-Ig that are functionally bivalent and readily blocked by soluble IgG, the B cell stimulating properties of SpA, which has five Ig-binding domains in tandem, can instead be significantly potentiated by soluble IgG (Figure 1).

### Soluble IgG is essential for the B-cell targeting activity of SpA

To investigate the influence of VH and IgG Fc regions, we generated a panel of recombinant monoclonal IgG with different Fab and Fc regions. This panel included recombinant IgG with Fab with a VHIII region, which displayed the expected Fab-mediated binding reactivity with native recombinant SpA (Supplementary Figure 1C). While those with a defined VHI-Fab did not interact with SpA via this domain (52) (Supplementary Figure 2A). These IgG were generated as whole molecules, with murine gamma constant regions of different subclasses, which varied in their capacity for Fc-mediated SpA binding interactions, as anticipated (Supplementary Table 1, Supplementary Figure 2B).

To assess for a possible influence of these soluble IgG on the *in vivo* superantigen properties of SpA, we utilized a well-proven adoptive cell transfer system. Here, donor splenic B cells from T15i (+/−) mice include ~20–30% B cells that express T15i-expressing B-cells, which can strongly interact with the Fab-binding site of SpA, and these are marked with the IgM^a^ allotype (53). The others are IgM^b^-bearing B cells with polyclonal repertoires that are little affected by SpA exposure (37, 45, 46, 56). With this system, the fate of T15i expressing B cells can be unambiguously tracked and quantified by flow cytometry (36, 43, 56).

**Figure 2:**
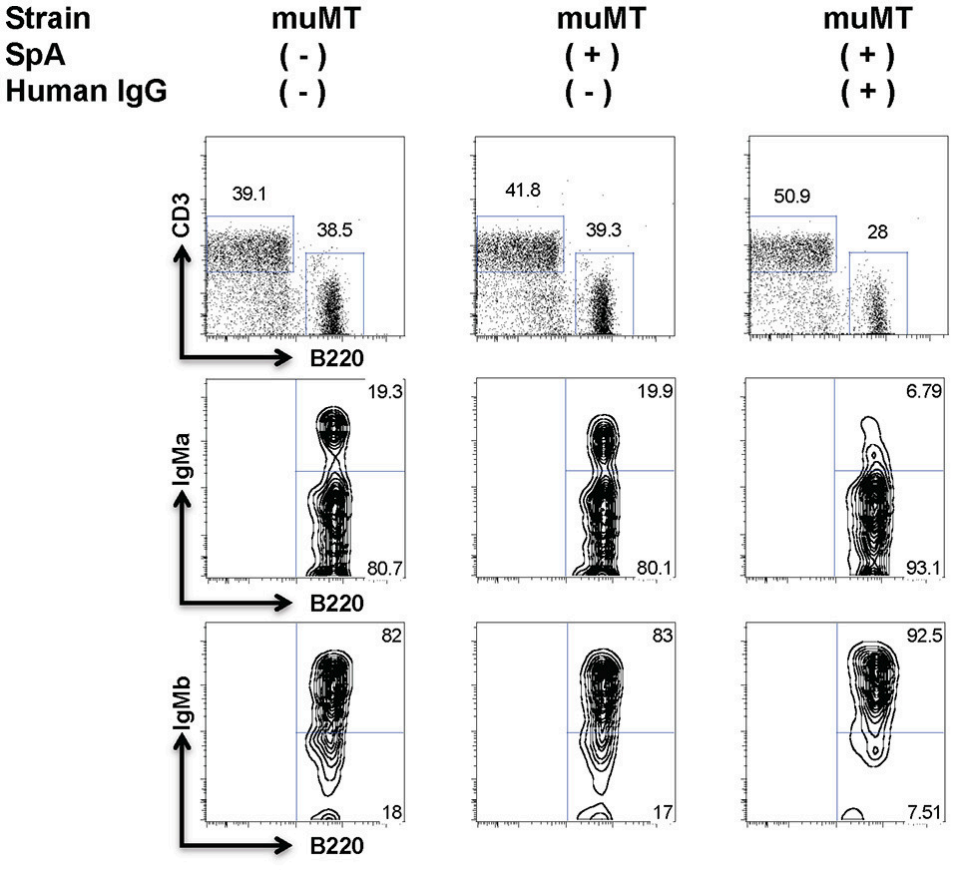
Soluble IgG is essential for the BCR targeted *in vivo* deletion induced by SpA. B-cell deficient (muMT) mice received adoptive transfer of T15i B cells, and after sham or human polyclonal IgG pretreatment were later challenged with PBS or SpA. Results represent residual splenic B220+ IgM^a^ + B cells, after flow cytometric gating on adoptively transferred CFSE-labeled mononuclear cells, which omits CD3+ cells. Treatment with hu IgG alone had no effect (not shown). Gating strategy was used to quantitate values shown in Figures 3, 5.

**Figure 3:**
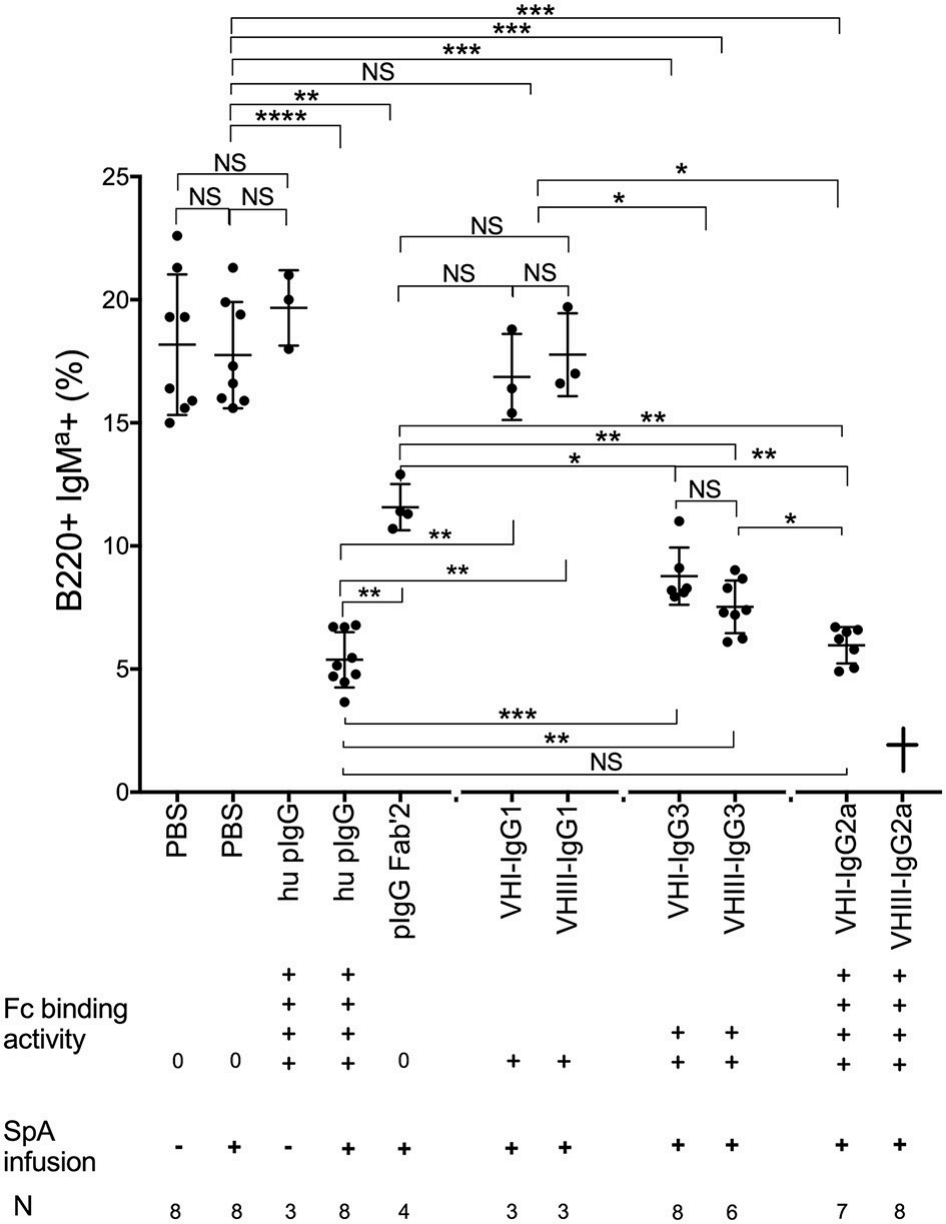
Extent of *in vivo* SpA-mediated B cell depletion reflects *in vitro* binding affinity. Assessment of SpA mediated depletion of transgenic T15i (+/−) B-cells following sham pretreatment or pretreatment with human polyclonal IgG (hu pIgG) or human polyclonal IgG F(ab)'2 (pIgG Fab'2) or monoclonal murine IgG, as indicated. At 72 h after SpA (or sham) treatment, levels of residual T15i B cells are indicated for individual mice. Results represent residual splenic IgM^a^ + B cells, after gating on adoptively transferred CFSE-labeled mononuclear cells, that identified only the B220+ IgM^a^+ Cells. After VHIII Fab IgG2a treatment, all mice died acutely (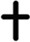) and residual B cells could therefore not be quantitated. Mann-Whitney test, *p < 0.05, ^**^p < 0.005, ^***^p < 0.001, ^****^p < 0.0001. NS, not significant. Binding activity data of murine IgG isotypes were from Wikipedia.

In mice that received sham saline infusions without Ig (as negative control treatments) or in mice treated with SpA alone without infused Ig there were no significant effects in the post-treatment representation of T15i B cells (Supplementary Figure 1)(Figures 2, 3), confirming earlier reports (43, 45, 46, 56). Yet compared to these control groups, mice that received both SpA and human polyclonal IgG displayed high-level VH targeted B-cell depletion (~70%) compared to baseline levels of these IgM^a^ marked T15i B cells (P<0.0001, two-tailed Fisher exact test) (Figures 2, 3). Hence, for the B-cell superantigen effect of SpA, our results confirm that soluble IgG play critical roles for efficient BCR cross-linking and induction of AICD (43), yet the contributions of Fc and Fab domains in polyclonal IgG were unclear.

### IgG subclass determines efficiency of B-cell deletion

To investigate the potential contributions of individual Fab-mediated and Fc-mediated binding interactions to SpA induced *in vivo* B-cell depletion, we studied the outcome of pretreatment exposures with different monoclonal IgG (Supplementary Figure 2). Amongst the panel of monoclonal IgG, treatment with mAbs of the IgG1 subclass, which has the weakest Fc-mediated binding interactions (38) (Supplementary Table 1) did not significantly deplete the targeted B cells (Figure 3). In fact, neither the VHI Fab IgG1 nor the VHIII Fab IgG1 mAb had an effect on these VHIII transgene-expressing B cells (Figure 3). By contrast, after pretreatment with IgG3 mAbs, SpA induced significantly greater VH targeted deletion of the adoptively transferred VHIII Fab-bearing T15i B cells (~60% depletion), which was only modestly but significantly less than the greater level of depletion associated with human polyclonal IgG pretreatment (~70%) (*p* = 0.0003) (Figure 3). Notably, of all mAbs the greatest depletion was induced by the VH1 Fab IgG2a that was equivalent to the level of depletion associated with infusions with human polyclonal IgG (Figure 3). We therefore concluded that SpA-IgG complex mediated deletion of B cells is not dependent on the Fab structure of the soluble IgG, whereas the subclass of the IgG constant region of the soluble IgG, which interact with the Fc-binding sites of native oligomeric SpA molecules (with relative activity of IgG2a Fc> IgG3 Fc> IgG1 Fc, Supplementary Table 1), determined the efficiency of B cell depletion. Unexpectedly in mice pretreated with VHIII Fab IgG2a, SpA rapidly induced death (discussed below), and therefore the efficiency of targeted B cell depletion could not be assessed. Cumulatively, whereas the Fc-binding sites of native oligomeric SpA were essential for the *in vivo* formation of these complexes, the Fab-binding sites of SpA mediated the VH specific interactions with the targeted B cells.

### SpA infusions can induce an anaphylactic-like fatal response

For the VHIII-Fab-IgG2a we were unable to assess the influence on B-cell depletion in our adoptive transfer system, as subsequent 1 mg (24 pmol) SpA infusions instead resulted in a rapid anaphylactic-like reaction in mice that displayed ataxia, tremors and impaired righting reflex, with death generally following within less than an hour. Indeed, even without adoptive transfer of T15i cells, mice pretreated with VHIII IgG2a infusions also suffered this fatal outcome after challenge with SpA. Moreover, in the presence of the endotoxin-free VHIII IgG2a, reduced challenges with 100 μg (2.4 pmol) SpA doses also acutely induced death. Whereas infusions of 10 μg (0.24 pmol) SpA doses did not cause a fatal reaction, this dose also caused little or no detectable depletion of adoptively transferred VHIII-bearing B cells (i.e., residual B cell levels were akin to those receiving sham infusions). In contrast, as described above, this SpA induced fatal outcome was neither induced by prior infusions of the VHI IgG2a, nor by IgG of other IgG subclasses, nor by human polyclonal IgG, and did not occur in the congenic strain that has only endogenous circulating IgG, even at higher SpA doses (45). These findings suggest that the formation of complexes between SpA with soluble monoclonal VHIII-Fab IgG2a molecules can result in fatal outcomes, potentially due to basophil and/or mast cell activation.

**Figure 4:**
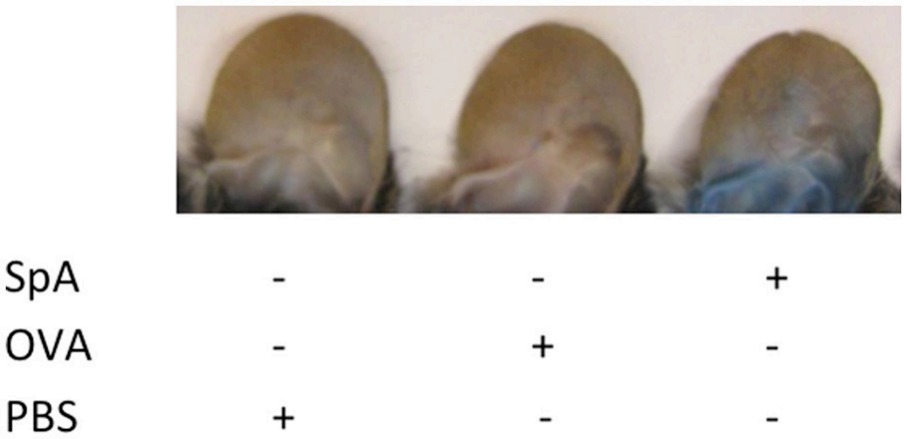
Evidence of plasma extravasation after SpA challenge in VHIII Fab-IgG2a treated mice. Wild type C57BL/6 mice received Evans Blue dye (0.5% w/v) then were infused with 1 mg VHIII IgG2a and 24 h later i.v. injection of 1 mg SpA or ovalbumin (OVA) or sham (PBS) treatment, as indicated. The ears of representative mice in each group are shown.

To determine whether these fatal responses were associated with systemic anaphylaxis (61), we repeated these SpA infusions in wild-type animals that had also received intravenous treatment with Evans blue dye, which has been used to detect increased vascular permeability associated with vascular injury (58). After SpA challenge, in mice that had been pretreated with VHIII-Fab IgG2a Evans blue dye was observable in the vascular compartment and diffused into the surrounding tissue, which was readily seen in the pinna of the ear (Figure 4). After control treatments, this dye was visible in the blood vessels alone (Figure 4), although this observation was admittedly descriptive and not otherwise quantitated. These findings therefore provide further evidence that both the Fab- and Fc-binding sites of SpA influence the functional properties of SpA-containing immune complexes. The concurrent exposure to spa and the VHIII-IgG2a, which displayed the strongest *in vitro* binding activities for the Fab- and Fc-binding sites of SpA, in fact triggered a fatal anaphylactic reaction, with features of cardiovascular collapse that is a common consequence of *S. aureus* bacteremia.

### Cellular Fc receptors and early complement are not required for SpA-mediated B-cell deletion

**Figure 5:**
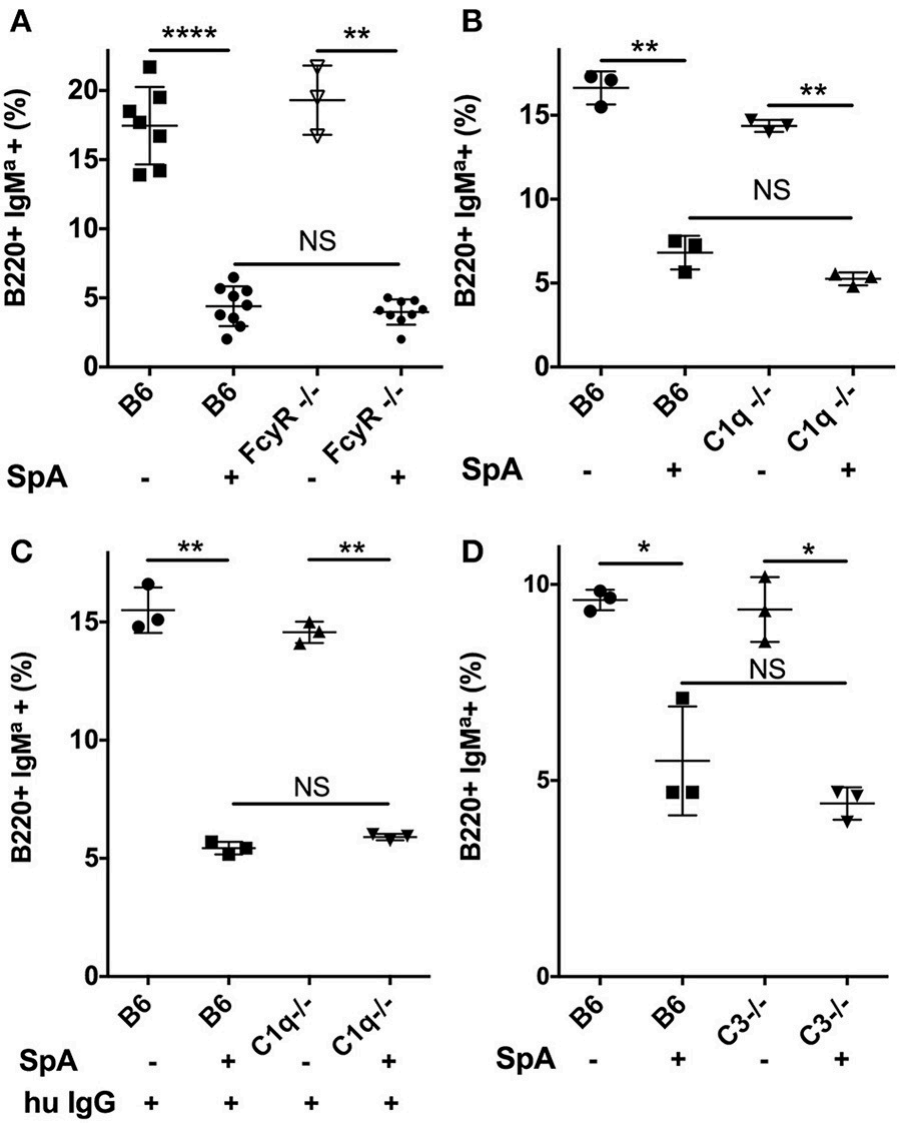
Neither early complement factors; C1q and C3, nor activating Fcγ receptors are required for SpA-mediated *in vivo* B-cell depletion. **(A)** *In vivo* responses of wildtype C57BL/6 (B6) or congenic Fc gamma deficient mice, without or with SpA challenge. **(B)** *In vivo* responses of wildtype B6 or congenic C1q- deficient mice, without or with SpA challenge. **(C)** After pretreatment with polyclonal human IgG, *in vivo* responses of wildtype B6 or congenic C1q- deficient mice, without or with SpA challenge. **(D)** *In vivo* responses of wildtype B6 or congenic C3- deficient mice, without or with SpA challenge. Splenic mononuclear cells from T15i+/− mice were CFSE labeled and then adoptively transferred into congenic mice, as indicated in each panel. Results reflect residual IgM^a^+ T15i transgenic B-cells after adoptive transfer and challenge. Mann-Whitney test, ^*^p < 0.05, ^**^p < 0.005, ^****^p < 0.0001. NS, not significant.

We also investigated whether the biological effects of SpA-IgG complexes were dependent on the effector functions of host cellular IgG-Fc binding domains (i.e., FcγR). We therefore performed T15i B cell adoptive cell transfers into recipient homozygotic Fcγ common chain knockout congenic mice (Figure 5A). Our results clearly demonstrated that host Fcγ-mediated effector mechanisms did not alter the superantigen effect of SpA on B-cell depletion (Figure 5A).

Immune complexes have the potential for the activation of complement via the classical pathway. Therefore, we next considered the potential contribution of complement activation to the B-cell death induced by SpA-containing complexes. *In vitro* studies have shown that complexes with murine IgG1 subclass antibodies generally have only weak capacity to activate the classical pathway of the complement cascade, while IgG2a or IgG3 subclass antibodies are much more potent (62). Furthermore, even though the constant regions of IgM cannot interact with SpA, IgM with VHIII-Fab can form complexes that also activate complement (63), which highlight that complement, which can mediate cell lysis, can be activated by SpA through different pathways.

We therefore repeated our *in vivo* challenge studies in homozygous C1q-deficient congenic mice, with the same SpA dose in mice that had only endogenous Ig, and we found that there was only a relatively lower efficiency (i.e., ~37%) of SpA-mediated B-cell depletion (Figure 5B). However, we were concerned that C1q gene deletion could affect immune development, which might also alter the composition or levels of circulating Ig. We therefore repeated these studies after pretreatment of C1q-deficient mice with human polyclonal IgG, and then SpA infusion resulted in ~70% B-cell deletion by SpA (Figure 5C) that was no different than the level of targeted B-cell depletion in complement-sufficient control mouse group (Figure 5).

To further investigate the possible contributions of complement, we next repeated these studies after adoptive transfer into C3-genetically deficient mice. Here, we saw no significant differences in the efficiency of SpA-mediated B-cell depletion in C3-deficient and in C3-sufficient wild-type congenic mice (Figure 5D). We also evaluated the effect on our model system after pretreatments with Cobra Venom Factor (CVF), which activates a convertase that degrades C3 *in vivo* (60). Yet CVF pretreatment also did not affect the outcome of SpA treatment on the efficiency of B-cell depletion (not shown). Cumulatively, these results showed that neither the classical pathway that is initiated by C1q, nor C3 that is at the confluence of the three major complement activation pathways, appear to play an essential role in SpA mediated B-cell depletion. Taken together with the above described results with FcγR deficient mice, neither complement dependent cytotoxicity that can result from downstream activation of the membrane attack complex that involves C5b-C9, nor FcγR responsible for antibody-dependent cellular cytotoxicity, appear to make essential contributions to SpA-Ig-mediated B-cell depletion.

### SpA production during systemic *S. aureus* infection

Whereas our above-described studies elucidated the essential requirements of soluble Ig for B-cell depletion, we also sought to quantitate the amounts of SpA that can be produced during an active experimental infection. We therefore performed infection studies, with inoculum doses confirmed to be sub-lethal, with the Newman strain of *S. aureus* in comparison with infections with an isogenic strain deleted for the *spa* gene (i.e., Δspa Newman), with mice sacrificed after 12 days.

In these mice, high levels of SpA were detectable in all tissues with abscesses that were examined (i.e., liver, kidney) (Supplementary Table 2). In representative B-cell deficient mice, we found more than 50 μg of total SpA in extracts of the largest visible abscesses in major organs. Based on the numbers of visible abscesses, we estimated that infection of a representative mouse induced a total yield of more than 200 μg of SpA. Notably, in equivalent studies in B-cell sufficient congenic C57BL/6 mice we also detected cumulative visible abscess but here the measurable SpA content was lower (total contents of ~14 μg of SpA) (unpaired two-tailed *t* test, *p* = 0.028). Albeit, this could reflect increased *in vivo* clearance or impaired detection of SpA detection due to formation of complexes with IgG. Hence, these studies documented that during experimental infection the cumulative amounts of SpA produced, could be equal to or exceed the SpA doses that cause *in vivo* VH targeted B-cell depletion (45).

## Discussion

In the current studies we have elucidated the nature of IgG complexes that are required for the superantigen properties of SpA. Firstly, the B-cell targeted effects of SpA have an absolute requirement for the recruitment of soluble IgG for BCR-mediated cellular activation, as documented by the measurement of intracellular calcium flux (Figure 1). Our data support the notion that these *in vivo* formed complexes enhance the BCR/co-receptor signal spreading that is responsible for SpA induced AICD (29).

Secondly, while B cells are clearly selectively targeted via the VHIII-restricted Fab binding site on SpA, the Fc-binding site of SpA was found to mediate interactions with soluble IgG, which presumably enhanced the functional valency of the complexes that formed. Indeed, the specific type of VH region in a full-length monoclonal IgG did not affect the efficiency of B-cell depletion effect (Figure 3). However, the outcome of *in vivo* exposure to SpA was dependent on the γ constant region subclass of the soluble IgG, which thereby indirectly affected the efficiency of B-cell depletion.

**Figure 6:**
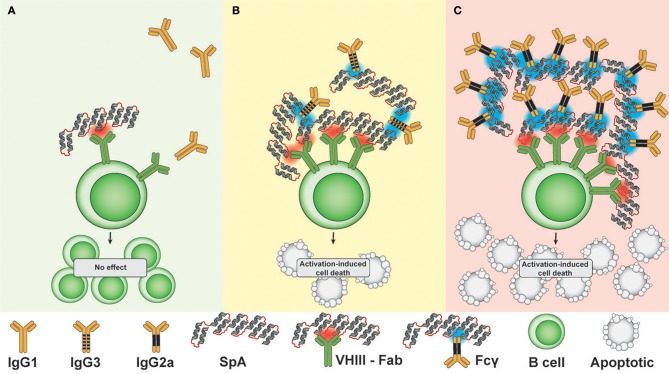
Mechanistic hypothesis: Lattice formation by SpA in complex with soluble IgG targets membrane-associated BCR to determine B cell fate. **(A)** In the presence of IgG capable of only very weak/undetectable Fc-mediated SpA binding (i.e., IgG1), the low effective valency of complexes does not result in depletion of VHIII Fab -BCR bearing B cells. **(B)** In the presence of IgG capable of medium level Fc-mediated SpA binding (i.e., IgG3), the greater effective valency of complexes causes cross-link VHIII BCR, resulting in a modest level of apoptotic depletion of VHIII-BCR bearing B cells. **(C)** In the presence of IgG capable of strong Fc-mediated SpA binding (i.e., IgG2a), B cell membrane-associated lattices are formed that cause high-level depletion of VHIII-BCR bearing B cells at 72 h after *in vivo* challenge, when second signal is insufficient. Red indicates a VHIII Fab-binding interaction with SpA, while blue indicates an Fc-mediated binding interaction with SpA.

Thirdly, we were surprised to discover that neither host cellular Fc receptors, which are responsible for antibody dependent cytotoxicity, nor complement pathways that lead to complement-dependent cytotoxicity, appeared to be essential for these SpA-mediated activities. Cumulatively, our studies have unraveled a seeming paradox regarding how this highly adapted toxin of an ubiquitous commensal-pathogen can effectively confound host adaptive immune defenses (Figure 6). However, other types of interactions with complement or Fc regions could still impinge on the availability of T-cell help in germinal centers or through other mechanisms.

Our studies rationalize how the SpA toxin can subvert an Achilles heel within host defenses that otherwise would enhance antibody-based defenses (28). In general, the activation of B lymphocytes requires effective cross-linking of membrane-associated BCR, either by multivalent antigens such as polysaccharides, or by aggregated antigens, which enable the formation of receptor-ligand lattices (64). While SpA has become the archetypic microbial Ig-binding protein, fundamental aspects of the structural bases for its B-cell targeted properties have remained enigmatic. Decades ago, Romagnani *et al*. reported that soluble natural SpA had little or no mitogenic activity *in vitro*, although after SpA was conjugated onto beads the increase of effective valency conveyed the capacity for inducing *in vitro* B-cell proliferation (65). In solution, the native SpA form takes on an extended conformation (49), and intravenous administration of SpA rapidly results in the *in vivo* formation of high molecular weight IgG-containing non-immune complexes, which persist for more than 24 h in the circulation (47). But these reports did not directly consider the contribution of the Fab- and Fc-binding sites on B cells.

From the perspective of the “two signal” model of lymphocyte activation (66), infusions of SpA that form an effective lattice with local IgG correlated with exuberant BCR signal 1 that is not counterbalanced by a sufficient signal 2 which as a consequence leads to rapid B-cell depletion (43) (discussed in Figure 6). In mice we have previously demonstrated that the mature B cell subsets, marginal zone and B-1 cells, have lower thresholds for SpA mediated death than naïve recirculating follicular B cells (i.e., B-2 cells) (56). Yet the *in vivo* implications of SpA exposure on human B-cell subpopulations during clinical *S. aureus* infections remain unclear, and our reductionist systems have not fully considered the influence of staphylococcal factors, such as enterotoxins or peptidoglycan that could induce inflammatory surrogates for signal 2 during clinical infection.

We have previously shown that the efficiency of *in vivo* depletion directly correlates with the valency and affinity of the Fab-binding interactions (43). While our crystallographic analyses have documented that a single domain of SpA can bind both a Fc molecule and VHIII-Fab molecule at once (40), our current results suggest that the actual *in vivo* complexes that cross-link membrane associated BCR ligand after SpA infusion involve both Fab- as well as Fc- mediated interactions that result in lattices with distinct biological properties. Our studies now demonstrate that the contributions of Fcγ domains of different subclasses of soluble IgG (Supplementary Table 1) also affects the properties of the SpA-containing lattices of these immune complexes.

To consider how our infusion model may be relevant to understanding the potential influence of SpA produced during clinical infection, we quantified the level of SpA in experimental abscesses. The total amount of SpA at harvest in a representative animal was estimated to at least equal the doses that cause targeted B-cell depletion in our infusion studies (45). In a recent report, the SpA released during experimental *S. aureus* infection in a mouse model was shown to induce responses that included high-levels of extra-follicular plasmablasts that peaked only at 6 weeks after inoculation (67). The potential effects of SpA on germinal center reactions and anamnestic responses have not yet been considered, although the above described findings do highlight the challenges inherent to fully characterizing the kinetics and anatomic distribution of the SpA that is released during infection. The simplistic approach we took represented a compromise, as it is difficult to experimentally measure local SpA concentrations at all sites where exposure of B cells can occur, and the total amount of SpA released over time is likely much greater than the amounts estimated to be contained in the abscesses (Supplementary Table 2).

Septic shock is an often-fatal condition evoked by a systemic inflammatory response to a pathogen. In the US, *S. aureus* is one of the most important causes of sepsis, which can be a sequella of bacteremia and infections in deep tissue with abscess formation, especially in kidneys and liver (68, 69). Multiple effectors have been implicated as contributors to septic shock, including leukocidin toxins and components of the gram-positive bacterial cell wall, such as lipoteichoic acids and peptidoglycans. Central roles for SpA have been suggested by reports that *S. aureus* mutants engineered to lack SpA have diminished virulence in both an intraperitoneal model and in an intravenous infection model associated with septic arthritis and renal abscess formation (70, 71). In our studies, the VHIII domain mediated strong Fab-mediated SpA binding, whereas the IgG2a Fc domain was associated with the strongest Fc-binding *in vitro* interactions. We therefore had anticipated that the VHIII-IgG2a would mediate the most complete *in vivo* B cell depletion. Yet, because these mice died after SpA challenge we could not measure the effect of VHIII-IgG2a on B cell depletion. Our investigations of this topic were limited, but the rapidly fatal outcome and evidence of increased vascular permeability suggested death was due to anaphylactic shock. We speculate that after *in vivo* formation of SpA complexes with the VHIII-IgG2a there was involvement of mast cells and basophils, which are known to bear the low affinity FcγRIIb (72), resulting in triggered cellular degranulation that contributed to anaphylaxis. In light of our findings regarding the cooperativity of the Fab- and Fc- binding sites of SpA, it is potentially relevant that most (i.e., 1, 2, and 4) human IgG subclasses mediate strong interactions with the Fc-binding site of SpA (Supplementary Table 1), which could also be involved in fatal clinical outcomes.

Antibodies expressing VHIII-Fab of other isotypes can also interact with SpA. Passive loading of such IgG or IgE onto host FcR result in arrays on the surfaces of non-lymphocyte leukocytes (e.g., mast cells, basophils), and has been postulated to mediate “superallergen” properties of SpA (73). As mentioned above, such interactions may contribute to adverse outcomes during infection, including septic shock. Whereas SpA interacts with a high frequency and range of VHIII gene-encoded antibodies, it is unclear if exposure to native SpA can induce responses to conventional epitopes on this toxin. However, by immunization with unnatural SpA forms with mutations that ablated the Ig-binding sites, Schneewind and co-workers have described non-VHIII encoded monoclonal antibodies that bind outside of the Fab-binding site (17, 74), which can protect neonatal mice from sepsis (75).

Patients with clinical *S. aureus* infections do not have global defects in the representation of recirculating anti-*S. aureus* memory B cells (10). However, from analyses of sorted activated plasmablasts, Pauli *et al*. reported that *S. aureus* infection is associated with VHIII-biased expansions of terminally-differentiated plasmablasts, but a paucity of plasmablasts to conventional staphylococcal antigens (76). Findings in pilot studies have suggested that i.v. SpA infusions in non-human primates can cause short-lived preferential expansions of VHIII-bearing CD27+ IgD- B cells (GJS and CSG, unpublished), which may reflect the lower activation thresholds of memory B cells (77). Given the importance of *S. aureus* infections as a public health threat, we believe that more complete investigations of the influence of SpA on the immune system during clinical infection are warranted.

In conclusion, our studies provide a mechanistic rationale for the superantigen properties of SpA, which may also represent a paradigm relevant to other microbial toxins with unconventional V region targeted activity in *S. aureus* (78) and other microbial commensal/opportunistic pathogens [reviewed in (41)]. Our studies support the notion that such toxins with superantigen properties may be highly effective at subverting host defenses. We also wonder whether during mucosal colonization and infection there is a subtle reprogramming of the human repertoire from these non-immune recurrent interactions with SpA.

## Ethics statement

All work with animals was performed with an approved protocol under the supervision of the NYUSOM Animal Care Program and IACUC.

## Author contributions

AU-M performed some experiments and aided in the preparing the first draft. CG performed some experiments and contributed to the design of other experiments and revision of the manuscript. GS contributed to all aspects of the work, from experimental design, manuscript preparation and revisions.

### Conflict of interest statement

The authors declare that the research was conducted in the absence of any commercial or financial relationships that could be construed as a potential conflict of interest.

## Supplementary material

Supplementary Table 1 IgG and subclass mediated SpA binding interactions.

Supplementary Table 2 SpA produced during *in vivo* experimental *S. aureus* infection.

Supplementary Figure S1 Gating strategy for identification and quantitation of adoptively transferred T15i (+/−) B-cells. **(A)** Side scatter and forward scatter. **(B)** Single cells to exclude duplexes. **(C)** Live/Dead, CFSE positive. **(D)** B220 positive CD3 negative. **(E)** B220 positive, IgM^a^ positive. **(F)** B220 positive IgM^b^ positive.

Supplementary Figure S2 Monoclonal and polyclonal IgG binding reactivity SpA forms and a control antigen. ELISA wells were precoated with **(A)** hen egg lysozyme (HEL), **(B)** chemically modified form of recombinant SpA (mSpA) that is devoid of Fc binding capacity, or **(C)** native recombinant SpA that has both Fc- and Fab- binding capacity. Reactivity of different murine monoclonal antibodies or human polyclonal IgG are depicted based on detection with HRP tagged anti-IgG reagents. The control VHI monoclonal IgG were generated with anti-HEL antibody genes with a J558/VHI clan region. The VHIII IgG expresses the classical T(EPC)15 antibody genes with a S107.1/VHIII clan region.
