# Supplementary material for: Essential Domain-Dependent Roles Within Soluble IgG for in vivo Superantigen Properties of Staphylococcal Protein A: Resolving the B-Cell Superantigen Paradox
Source: Front Immunol. 2018 Sep 19;9:2011. doi: 10.3389/fimmu.2018.02011 (PMC6156153; doi:10.3389/fimmu.2018.02011)
Supplement: Supplementary Table 1 — IgG and subclass mediated SpA binding interactions. [file Table_1.PDF]

Supplementary Table 1. IgG and subclass mediated SpA binding interactions

Species	Isotype/subclass	Fc mediated binding of SpA
Human	Polyclonal	++++
	IgG1	++++
	IgG2	++++
	IgG3	-
	IgG4	++++
	IgM	-
	IgA	-
	IgE	-
Mouse	IgG1	+
	IgG2a	++++
	IgG2b	+++
	IgG3	++
Rabbit	polyclonal IgG	++++

Adapted from (Protein A, Wikipedia).
