# Supplementary material for: Essential Domain-Dependent Roles Within Soluble IgG for in vivo Superantigen Properties of Staphylococcal Protein A: Resolving the B-Cell Superantigen Paradox
Source: Front Immunol. 2018 Sep 19;9:2011. doi: 10.3389/fimmu.2018.02011 (PMC6156153; doi:10.3389/fimmu.2018.02011)
Supplement: Supplementary Table 2 — SpA produced during in vivo experimental S. aureus infection. [file Table_2.PDF]

Supplementary Table 2. SpA produced during *in vivo* experimental *S. aureus* infection.

Host	*S. aureus* strain	Organ	Total abscess weight (mg)	ng (fmol) SpA) / mg abscess	Total SpA µg (pmol)
µMT	Newman	Kidney	53.4	415.2 (9.88)	22.2 (0.528)
µMT	Newman	Kidney	57.0	148.4 (3.57)	8.5 (0.202)
µMT	Newman	Liver	52.5	347.9 (8.28)	18.3 (0.436)
µMT	Newman	Liver	41.4	57.7 (1.37)	2.4 (0.057)
B6	Newman	Kidney	48.0	4.7 (0.11)	0.2 (0.005)
B6	Newman	Kidney	34.8	39.1 (0.93)	1.4 (0.033)
B6	Newman	Liver	64.1	19.0 (0.45)	1.2 (0.029)
B6	Newman	Liver	68.7	25.9 (0.62)	1.8 (0.043)
µMT	Δ spa	Kidney	38.3	ND	ND
µMT	Δ spa	Kidney	16.1	ND	ND
