# Supplementary material for: Essential Domain-Dependent Roles Within Soluble IgG for in vivo Superantigen Properties of Staphylococcal Protein A: Resolving the B-Cell Superantigen Paradox
Source: Front Immunol. 2018 Sep 19;9:2011. doi: 10.3389/fimmu.2018.02011 (PMC6156153; doi:10.3389/fimmu.2018.02011)
Supplement: Supplementary Figure S1 — Gating strategy for identification and quantitation of adoptively transferred T15i (+/−) B-cells. (A) Side scatter and forward scatter. (B) Single cells to exclude duplexes. (C) Live/Dead, CFSE positive. (D) B220 positive CD3 negative. (E) B220 positive, IgMa positive. (F) B220 positive IgMb positive. [file Image_1.PDF]

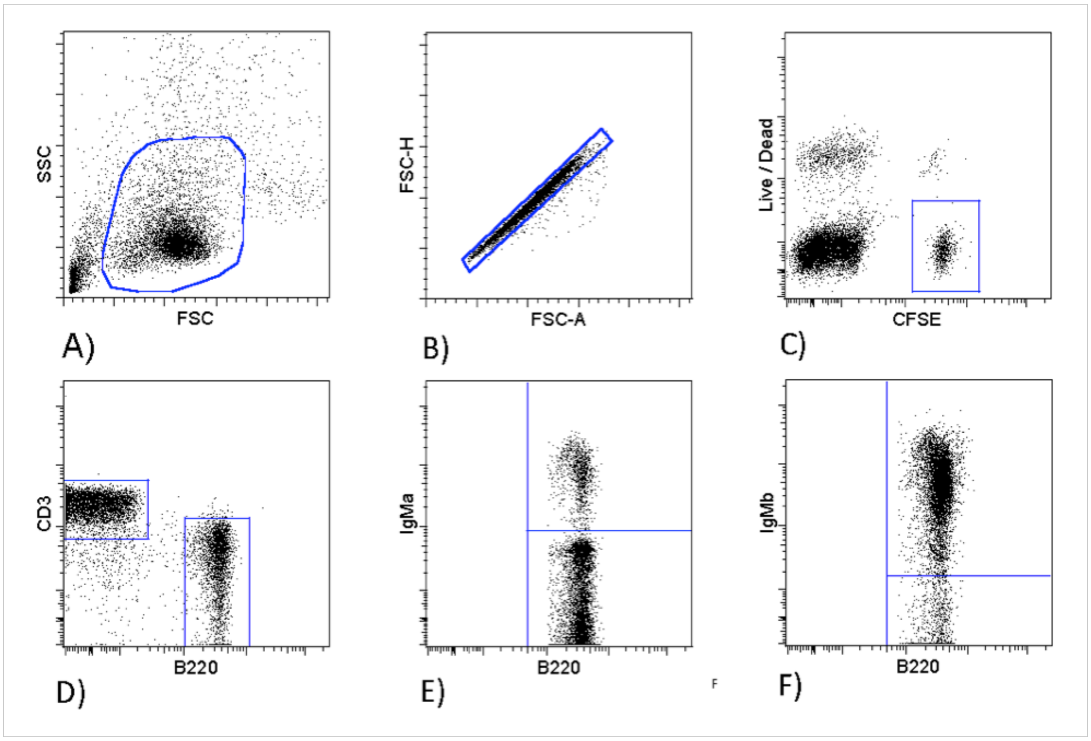

Supplementary Figure 1. Gating strategy for identification and quantitation of adoptively transferred T15i (+/-) B-cells. A) Side scatter and forward scatter. B) Single cells to exclude duplexes. C) Live/Dead, CFSE positive. D) B220 positive CD3 negative. E) B220 positive, IgM^a positive. F) B220 positive IgM^b positive.
