# Supplementary figures and images for: Essential Domain-Dependent Roles Within Soluble IgG for in vivo Superantigen Properties of Staphylococcal Protein A: Resolving the B-Cell Superantigen Paradox
Source: Front Immunol. 2018 Sep 19;9:2011. doi: 10.3389/fimmu.2018.02011 (PMC6156153; doi:10.3389/fimmu.2018.02011)

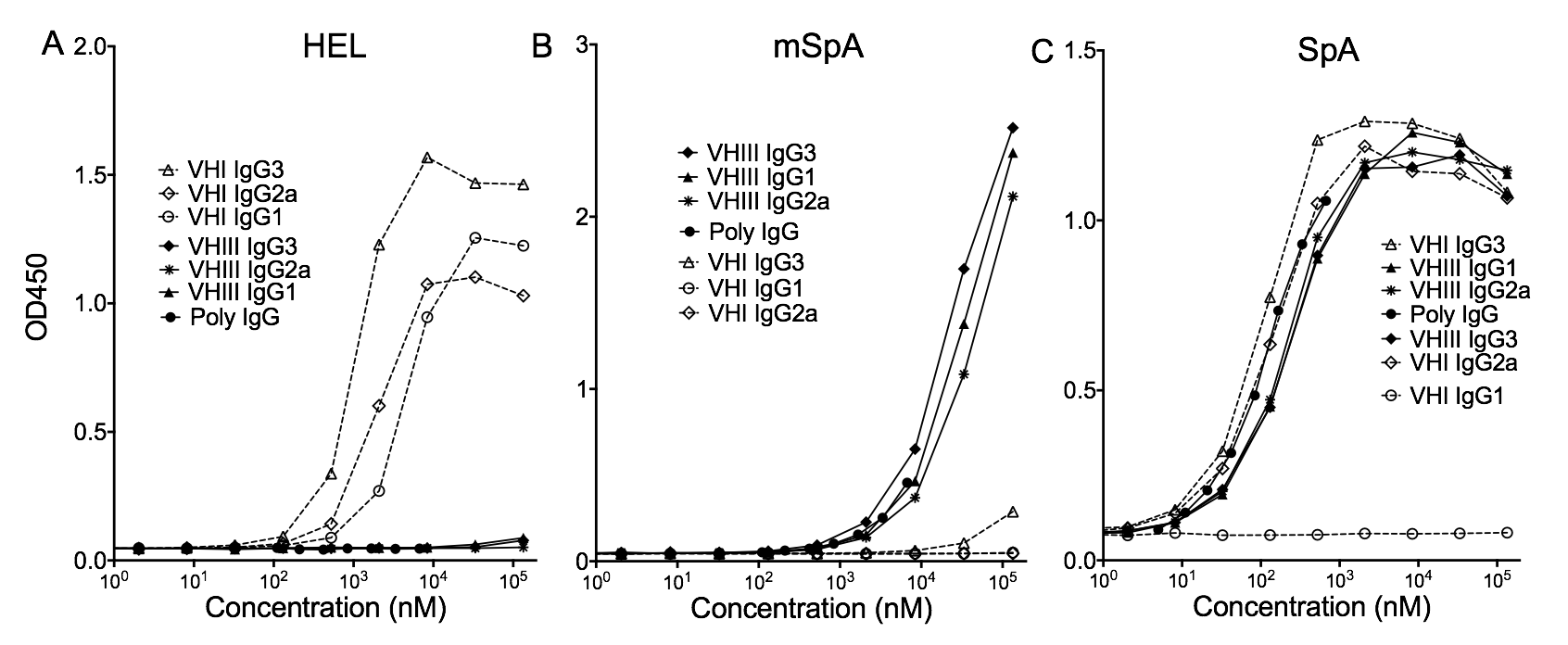

Supplement: Supplementary Figure S2 — Monoclonal and polyclonal IgG binding reactivity SpA forms and a control antigen. ELISA wells were precoated with (A) hen egg lysozyme (HEL), (B) chemically modified form of recombinant SpA (mSpA) that is devoid of Fc binding capacity, or (C) native recombinant SpA that has both Fc- and Fab- binding capacity. Reactivity of different murine monoclonal antibodies or human polyclonal IgG are depicted based on detection with HRP tagged anti-IgG reagents. The control VHI monoclonal IgG were generated with anti-HEL antibody genes with a J558/VHI clan region. The VHIII IgG expresses the classical T(EPC)15 antibody genes with a S107.1/VHIII clan region. [file Image_2.TIFF]
